# Integrated transcriptomic, molecular docking, and mendelian randomization analysis reveal a microbial–propionate–JUN pathway in renal ischemia-reperfusion injury

**DOI:** 10.1080/21505594.2026.2673655

**Published:** 2026-05-14

**Authors:** Jun Li, Ruizhen Huang, Xing Wang, Yunfeng Zhang, Zuhuan Xu, Penglin Zhang, Honglin Hu

**Affiliations:** Department of Urology, The Second Affiliated Hospital, Jiangxi Medical College, Nanchang University, Nanchang, China

**Keywords:** Renal ischemia-reperfusion injury, gut–kidney axis, short-chain fatty acids, molecular docking, mendelian randomization

## Abstract

Renal ischemia–reperfusion injury (IRI) is a major cause of acute kidney injury and is characterized by oxidative stress, immune cell infiltration, and inflammatory signaling activation. Although gut microbiota and their metabolites, especially short-chain fatty acids, are involved in systemic immune regulation, their role in renal IRI remains unclear. Here, we integrated transcriptomic analysis, gut-derived metabolite target prediction, molecular docking, and Mendelian randomization (MR) to explore potential microbiota–metabolite–host regulatory mechanisms in renal IRI. We identified 32 target genes of gut-derived metabolites using the gutMGene, Similarity Ensemble Approach, and SwissTargetPrediction databases. In two renal IRI datasets (GSE126805 and GSE90861), 263 and 641 differentially expressed genes were identified, respectively, and hub genes were mainly enriched in the TNF and IL-17 signaling pathways. Among them, JUN was identified as a key hub linking gut microbiota-associated metabolites to renal inflammatory signaling. MR analysis showed that *Akkermansia muciniphila* was positively associated with kidney injury susceptibility [*p* = 0.026, odds ratio (OR) = 1.219], whereas *Ruminococcus bromii* was negatively associated with kidney injury susceptibility (*p* = 0.011, OR = 0.740). Because propionate, rather than butyrate, was the shared metabolite associated with both taxa, subsequent analyses focused on the propionate–JUN interaction. Molecular docking and dynamics supported strong binding between propionate and JUN. Overall, these findings suggest a potential gut–kidney regulatory axis involving *A. muciniphila/R. bromii*–propionate–JUN–TNF/IL-17 signaling in renal IRI, providing new insight into microbiota-associated mechanisms of renal inflammatory injury.

## GRAPHICAL ABSTRACT

**Figure:**
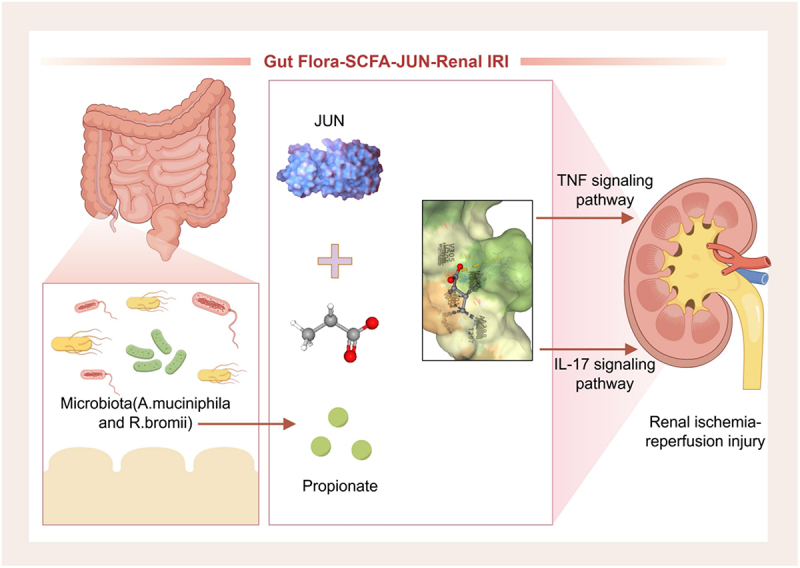

## Introduction

Renal ischemia-reperfusion injury (IRI) is an important pathological basis of acute kidney injury (AKI), a common and serious complication in surgery, organ transplantation and critical care medicine, with high clinical mortality and disability rates [1–3]. The pathogenesis of IRI is multifactorial, involving abrupt fluctuations in oxygen supply, mitochondrial dysfunction, and amplification of inflammatory signaling cascades, all of which contribute to tubular epithelial damage and hinder renal recovery [4–6]. Central to this process is the activation of endogenous inflammatory transcriptional programs, which drive the secretion of pro-inflammatory cytokines, immune cell infiltration, and regulated cell death. However, the regulatory factors upstream of these transcriptional programs, especially exogenous signals originating from the intestinal microcosm, have so far lacked systematic elucidation.

Recent studies have highlighted the gut microbiota as a systemic modulator of host immunity and organ function [7,8]. Among microbial-derived metabolites, short-chain fatty acids (SCFAs) such as acetate, butyrate, and propionate have been shown to exert anti-inflammatory, antioxidant, and barrier-preserving effects across multiple tissues [9,10]. SCFAs act not only as metabolic substrates but also as signaling molecules that influence host targets, including transcription factors, epigenetic enzymes, and G protein – coupled receptors [11,12]. Although the immunoregulatory roles of SCFAs are well established, whether they participate directly in renal IRI by modulating inflammatory transcriptional programs remains to be elucidated.

The transcription factor JUN represents a potential mechanistic link in this context. As a key component of the Activator Protein-1 (AP-1) complex, JUN is rapidly upregulated in response to stress signals and orchestrates the expression of genes involved in cytokine production, apoptosis, and tissue remodeling [13]. Sustained JUN activation has been observed in IRI models of multiple organs, including the kidney and brain [14,15]. However, its upstream regulatory landscape remains largely undefined. Given the ability of SCFAs to influence transcription factor activity through receptor-mediated or epigenetic pathways, we hypothesized that propionate may directly target JUN, thereby modulating its activation during renal IRI.

**Figure 1.**
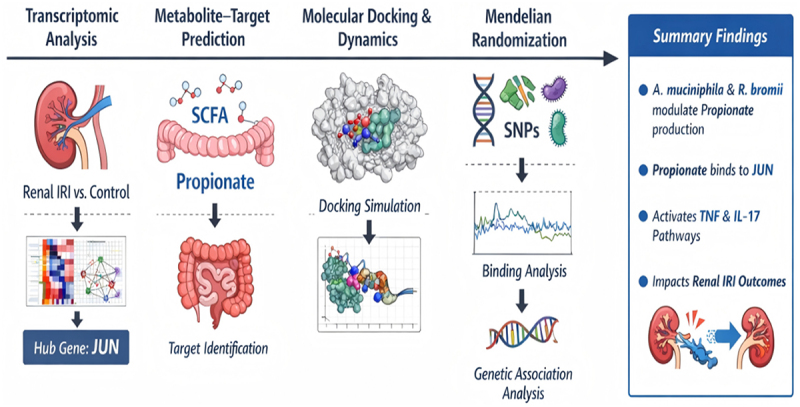
Workflow of the integrative multi-omics analysis in this study.

To test this hypothesis, we employed a multi-omics strategy combining transcriptomic analysis, metabolite – target prediction, molecular docking simulation, and Mendelian randomization (MR) to construct and validate a regulatory axis linking gut microbiota – derived propionate to JUN in renal IRI. Our findings reveal that *Akkermansia muciniphila and Ruminococcus bromii*, through their roles in mucin degradation and resistant starch metabolism, modulate SCFA production and influence systemic propionate availability. Propionate showed favorable structural interaction with JUN and regulates its downstream activation of TNF and IL-17 signaling pathways, thereby potentially influencing renal inflammatory injury in the context of renal IRI. This study uncovers a novel microbial – host regulatory mechanism by which gut-derived metabolites influence inflammatory transcriptional responses and provides a theoretical foundation for microbiota-targeted interventions in AKI. The overall study design and analytical workflow are summarized in Figure 1.

## Materials and methods

### Identification of the gut microbiota and its metabolites and targets

We retrieved data on gut microbiota-derived metabolites and their human targets from the gutMGene v2.0 database (http://bio-computing.hrbmu.edu.cn/gutmgene/#/Home) [16]. Corresponding Simplified Molecular Input Line Entry System (SMILES) formats for each metabolite were acquired from the PubChem database (https://pubchem.ncbi.nlm.nih.gov/). Potential target genes of these metabolites were then predicted using the Similarity Ensemble Approach (SEA) (https://sea.bkslab.org/) and SwissTargetPrediction (STP) (http://www.swisstargetprediction.ch/) platforms, with the species parameter set to Homo sapiens. The overlapping targets from gutMGene, SEA, and STP databases were identified via Venn diagrams and selected for downstream analysis as consensus gut-derived targets.

### Acquisition of renal IRI-associated genes

Transcriptomic datasets related to renal IRI were downloaded from the Gene Expression Omnibus (GEO) database (https://www.ncbi.nlm.nih.gov/geo/), including GSE90861 and GSE126805. Raw microarray data and annotation files were obtained directly from GEO. The GSE90861 dataset includes 23 IRI samples and 23 pre-injury controls [17], while GSE126805 consists of 42 IRI samples and 41 controls [18]. Each dataset was processed independently. Probe annotation files provided by GEO were used to map probes to official gene symbols; when multiple probes mapped to the same gene, the average expression value was used. Expression matrices were normalized within each dataset prior to differential expression analysis. Because the two cohorts were not merged for joint modeling, cross-dataset batch-effect correction was not applied; instead, differential expression analysis was performed separately for each dataset and overlapping differentially expressed genes (DEGs) were identified for downstream analysis. Differential gene expression analysis was conducted using the R package limma, and genes meeting the criteria of |log2 fold change| >1 and adjusted *p* < 0.05 were defined as DEGs. A Venn diagram was constructed via Venny 2.1 to identify overlapping DEGs, which were considered IRI-related target genes.

### Protein-protein interaction (PPI) network and hub gene identification

To explore interactions among IRI-related genes, a PPI network was built using the STRING database (https://cn.string-db.org/) with default confidence parameters. The resulting network was visualized in Cytoscape, and key gene modules were identified using the Molecular Complex Detection (MCODE) plugin. Hub genes were subsequently ranked and selected using the Maximal Clique Centrality (MCC) algorithm in the cytoHubba plugin, and the top 10 were retained for further analysis.

### Functional enrichment analysis of hub genes

Gene Ontology (GO) analysis was conducted to classify the biological processes (BP), cellular components (CC), and molecular functions (MF) associated with 10 hub genes. Kyoto Encyclopedia of Genes and Genomes (KEGG) pathway enrichment analysis was also performed to identify associated signaling pathways. Multiple-testing correction was conducted separately for each enrichment analysis, with GO and KEGG results adjusted independently using Bonferroni correction. Terms or pathways with adjusted *p* < 0.05 were considered statistically significant. GO and KEGG enrichment results were visualized using bubble plots.

### Construction of the gut microbiota–metabolite–target–pathway–disease (G-M-T-P-D) network

To dissect the integrative relationship between microbiota, metabolites, targets, signaling pathways, and disease phenotypes, a G-M-T-P-D network was constructed. Common targets shared between microbial metabolites and renal IRI were first identified. KEGG enrichment was then used to select the top 10 significantly enriched pathways. These pathways were linked to key microbial metabolites and upstream microbial taxa. Cytoscape v3.10.2 was used to visualize and build the comprehensive network.

### MR analysis of gut microbial traits and AKI susceptibility

Because genome-wide association study (GWAS) summary statistics specifically for renal IRI are not yet available at a sufficiently large scale, AKI was adopted as a clinically relevant proxy outcome in the MR analysis. This substitution was considered justifiable because renal IRI is a major mechanistic driver of AKI in several clinical contexts, especially kidney transplantation, major surgery, and critical illness. In addition, renal IRI and AKI share core pathobiological features, including tubular epithelial damage, oxidative stress, mitochondrial dysfunction, microvascular disturbance, and inflammatory activation. However, AKI is a broader and more heterogeneous clinical syndrome than renal IRI. Therefore, the MR results should be interpreted cautiously as providing evidence for the relevance of the identified gut microbial signals to kidney injury susceptibility, rather than definitive evidence of causal specificity for renal IRI itself.

To investigate the potential relationship between gut microbial traits and kidney injury susceptibility, a two-sample bidirectional MR framework was applied. Summary statistics for gut microbiota were obtained from the Dutch Microbiome Project, which included 7,738 European participants and profiled 207 microbial taxa together with 205 metabolic pathways [19]. In accordance with the aims of the present study, single-nucleotide polymorphisms (SNPs) associated with 207 gut microbial taxa (study accession numbers: GCST90027808–GCST90027707) were extracted as instrumental variables (IVs). These SNPs represent host genetic variants associated with the abundance of specific gut microbial taxa, rather than genetic variants derived from the microorganisms themselves. Therefore, this MR framework does not imply that gut microbiota directly cause renal ischemia itself, but rather evaluates whether host genetic predisposition to specific gut microbial traits is associated with susceptibility to AKI.

SNPs reaching the conventional genome-wide significance threshold (*p* < 5 × 10^-8) were preferentially selected. When the number of eligible IVs was limited, a relaxed threshold (*p* < 1 × 10^-5) was adopted, together with linkage disequilibrium clumping criteria of r^2^ <0.001 and a 10,000 kb window. To minimize weak instrument bias, only IVs with an F-statistic >10 were retained [20,21]. GWAS summary statistics for AKI were obtained from the FinnGen Consortium (FinnGen ID: finn-b-N14_ACUTERENFAIL), including 2,383 cases and 212,841 controls. MR analysis were performed using the TwoSampleMR package in R. The inverse variance weighted (IVW) method was used as the primary estimator of causal effect, while MR-Egger, weighted median, and weighted mode methods were applied as complementary approaches. Sensitivity analysis included reverse MR, the MR-Egger intercept test for horizontal pleiotropy, and Cochran’s Q test for heterogeneity [22,23]. In addition, Bayesian weighted Mendelian randomization (BWMR) was used to further assess the robustness of the results. A *p* value < 0.05 was considered statistically significant.

### Molecular docking between metabolites and core genes

To assess direct interactions between microbial metabolites and core gene products, molecular docking simulations were conducted. The crystal structure of the human AP-1 complex [Protein Data Bank (PDB) ID: 1JUN] was retrieved from PDB database (https://www.rcsb.org/), containing JUN (chain A), FOS (chain B), and DNA (chain C). Only chain A (JUN) was retained using PyMOL (v2.5.2); chains B and C were removed. The cleaned protein structure was protonated and energy-minimized for docking. Metabolite three dimensional (3D) structures were generated from SMILES via PubChem, converted using Open Babel, and similarly minimized. AutoDock Vina was used for docking within a defined grid box centered around known or predicted active sites. Binding energies (kcal/mol) were computed, and optimal binding poses were visualized using PyMOL and Discovery Studio to analyze key molecular interactions.

### Molecular dynamics simulation

Molecular dynamics simulation was performed to investigate the stability and interaction dynamics of protein complexes with butyrate and propionate. Each system was simulated for 100 ns under standard conditions. The trajectories were analyzed to calculate root mean square deviation (RMSD) of the protein backbone, ligand, and complex to assess structural stability over time. Residue-level flexibility was evaluated using root mean square fluctuation (RMSF). The compactness of the protein structure was characterized by the radius of gyration (Rg), while solvent-accessible surface area (SASA) was computed to estimate changes in solvent exposure during the simulation. Protein – ligand interactions were further analyzed by monitoring the number of hydrogen bonds throughout the trajectory.To explore the conformational space, free energy landscapes (FELs) were constructed using RMSD and Rg as collective variables, allowing identification of energetically favorable conformational states.

### Statistical analysis

All statistical analysis were performed using R software (version 4.2.2). DEGs were identified using the limma package. GO and KEGG enrichment analysis were performed using clusterProfiler, with Bonferroni correction applied for multiple testing; adjusted *p* < 0.05 was considered statistically significant. MR analysis were conducted using the TwoSampleMR package, with the IVW method as the primary approach and MR-Egger, weighted median, and weighted mode methods as complementary analysis. Sensitivity analysis included the MR-Egger intercept test, Cochran’s Q test, reverse MR, and BWMR. A threshold of *p* < 0.05 was considered statistically significant.

## Results

### Identification of gut microbiota metabolites and their target genes

**Figure 2.**
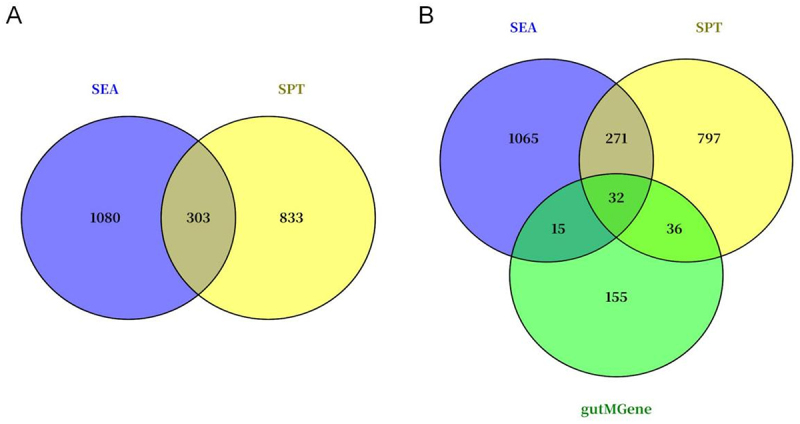
Identification of gut microbiota metabolite targets. (A) Common gut microbiota metabolite targets in sea and spt databases; (B) Common targets for gut microbiota metabolite targets and human gut targets. Similarity Ensemble Approach, sea; SwissTargetPrediction, STP.

We initially obtained 277 gut microbiota-derived metabolites and 238 human gut-related target genes from the gutMGene v2.0 database (Supplementary Table S1). Subsequently, 1,383 and 1,136 targets related to these metabolites were identified using the SEA and STP databases, respectively (Supplementary Table S1). A total of 303 overlapping targets between SEA and STP were regarded as primary metabolite targets (Figure 2(A)). Finally, 32 genes that overlapped among the gutMGene, SEA, and STP databases were defined as core targets of gut microbial metabolites (Figure 2(B)).

### Identification of renal IRI-Associated target genes

**Figure 3.**
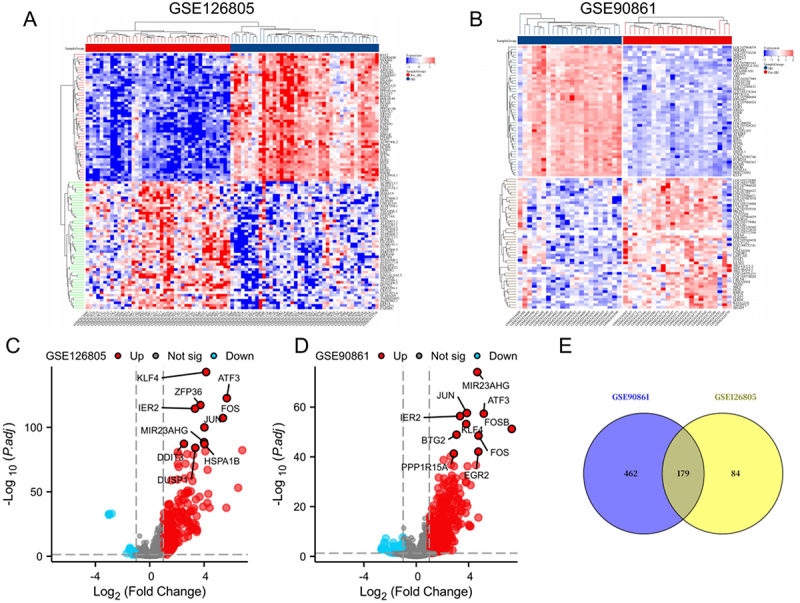
Common DEGs identified by renal ischemia-reperfusion injury expression profiling. (A, B) Heatmaps of the first 100 DEGs in GSE126805 and GSE90861; (C, D) Volcano plots of the DEGs in the GSE126805 and the GSE90861 dataset; (E) Venn diagram showing that 179 DEGs in the two datasets overlapping. Differentially expressed gene, deg.

To elucidate transcriptional changes associated with renal IRI, we analyzed two publicly available expression datasets: GSE126805 and GSE90861, both comparing gene expression profiles between IRI and control kidney tissues. Hierarchical clustering heatmaps (Figure 3(A,B)) showed clear expression pattern distinctions between groups in both datasets. Volcano plots illustrated significantly DEGs (Figure 3(C,D)). A total of 263 DEGs were identified in GSE126805 (Supplementary Table S2), including 242 upregulated and 21 downregulated genes (adjusted *p* < 0.05, |log_2_FC| >1), such as key inflammatory transcription factors JUN, ATF3, FOS, and ZFP36. Similarly, in GSE90861, 641 DEGs were detected, including 575 upregulated and 66 downregulated genes, with significant upregulation of JUN, FOS, ATF3, and IER2 (Supplementary Table S2). A Venn diagram analysis (Figure 3(E)) revealed 179 overlapping DEGs across the two datasets, which were selected for further investigation as IRI-related target genes.

### Identification and functional enrichment of core genes in renal IRI

**Figure 4.**
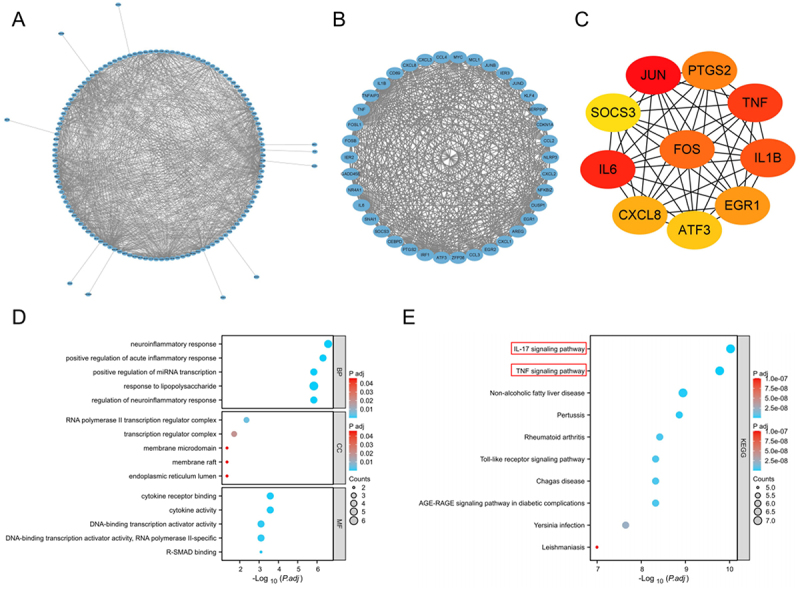
Analysis of core gene identification and functional enrichment in renal ischemia-reperfusion injury. (A) Complete PPI network constructed from overlapping differentially expressed genes. (B) Highly interconnected core modules extracted from the PPI network demonstrating the dense interplay structure of key inflammatory factors. (C) The top 10 core genes identified based on the cytoHubba plugin. (D) GO enrichment analysis of the 10 core genes. (E) KEGG enrichment analysis of 10 core genes. Protein-Protein Interaction, PPI; Gene Ontology, GO; Kyoto Encyclopedia of Genes and Genomes, KEGG.

To further explore the functional relevance of overlapping DEGs in IRI, we constructed a PPI network and performed functional enrichment analysis. The PPI network was generated using STRING and visualized in Cytoscape (Figure 4(A)). Highly connected modules within the network were identified using the MCODE plugin (Figure 4(B)). Through cytoHubba, the top 10 hub genes were determined (Figure 4(C)): JUN, FOS, TNF, IL6, PTGS2, CXCL8, IL1B, ATF3, EGR1, and SOCS3, many of which are closely associated with acute inflammation and immune responses. GO analysis revealed enrichment in BP (neuroinflammatory response, positive regulation of acute inflammatory response, response to lipopolysaccharide), CC (transcription regulator complex, membrane raft), and MF (cytokine receptor binding, transcription factor activity). KEGG pathway analysis showed significant enrichment in classical immune-inflammatory signaling cascades, particularly the IL-17 and TNF signaling pathways. The top enriched GO terms and KEGG pathways are shown using bubble plots (Figure 4(D,E)). The significance values displayed in the plots represent Bonferroni-adjusted *p* values, and the full enrichment results are provided in Supplementary Table S3.

### G-M-T-P-D network construction and molecular docking

**Figure 5.**
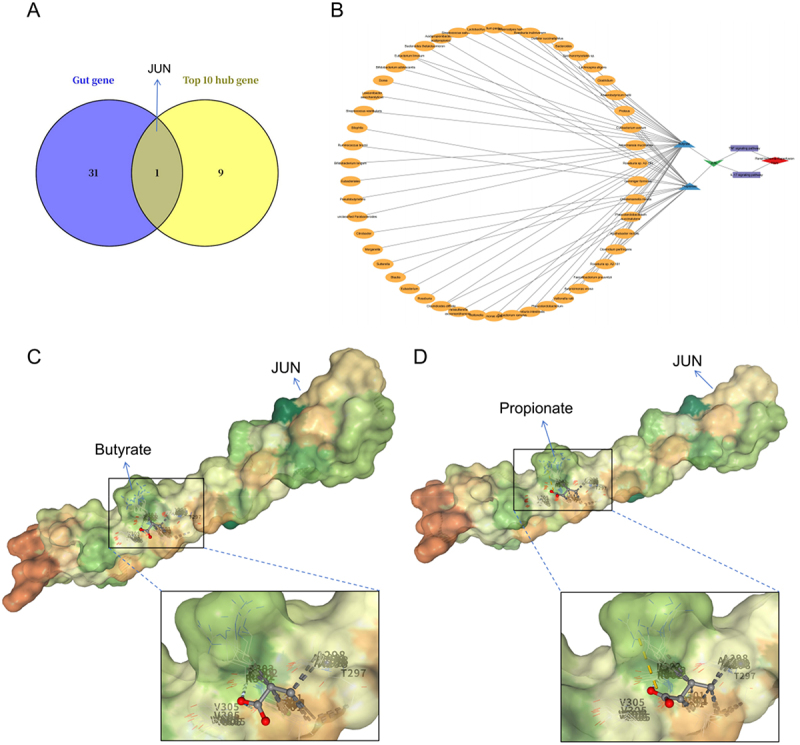
JUN as a gut-derived regulatory target can be regulated by binding of SCFAs. (A) Venn diagram showing JUN as the only overlapping gene belonging to both gut-derived targets and core PPI genes. (B) The “gut microbiota-metabolite-target-pathway-disease” network centered on JUN. (C, D) Molecular docking model of JUN protein with butyrate and propionate. Short-chain fatty acid, SCFA; Protein-Protein Interaction, PPI.

To explore potential regulatory interactions between gut microbial metabolites and core IRI-related genes, we intersected gut-derived targets with PPI results. As shown in the Venn diagram (Figure 5(A)), JUN was the only hub gene that overlapped both as a metabolite target and as one of the top 10 IRI-related hub genes, indicating its possible role as a key effector linking gut microbiota to renal inflammation. A G-M-T-P-D network was constructed (Figure 5(B)), illustrating how metabolites such as butyrate and propionate could regulate JUN activity, thereby influencing downstream IL-17 and TNF signaling and the progression of IRI. Additionally, we identified 50 bacterial taxa potentially responsible for producing butyrate and propionate for subsequent comparison.

Molecular docking was performed to validate interactions between JUN (PDB ID: 1JUN) and these SCFAs. Butyrate bound JUN with a binding energy of −13.4 kcal/mol, forming stable hydrogen bonds with residues T297, V303, V305, and A298 (Figure 5(C), Table 1). Propionate also exhibited stable binding (−12.1 kcal/mol) with similar interaction sites (Figure 5(D), Table 1). These interactions were localized to critical domains of JUN, suggesting that SCFAs may directly modulate JUN activity and downstream transcriptional regulation of inflammatory genes.

**Table 1.** Molecular docking information of JUN proteins with metabolites butyrate and propionate.

					Grid box centre		
Protein	PDB ID	Metabolite	CID	Binding energy (kcal/mol)	X	Y	Z
JUN	1JUN	Butyrate	104,775	−13.4	4	−11	−2
		Propionate	104,745	−12.1	4	−11	−2

### Molecular dynamics simulation

**Figure 6.**
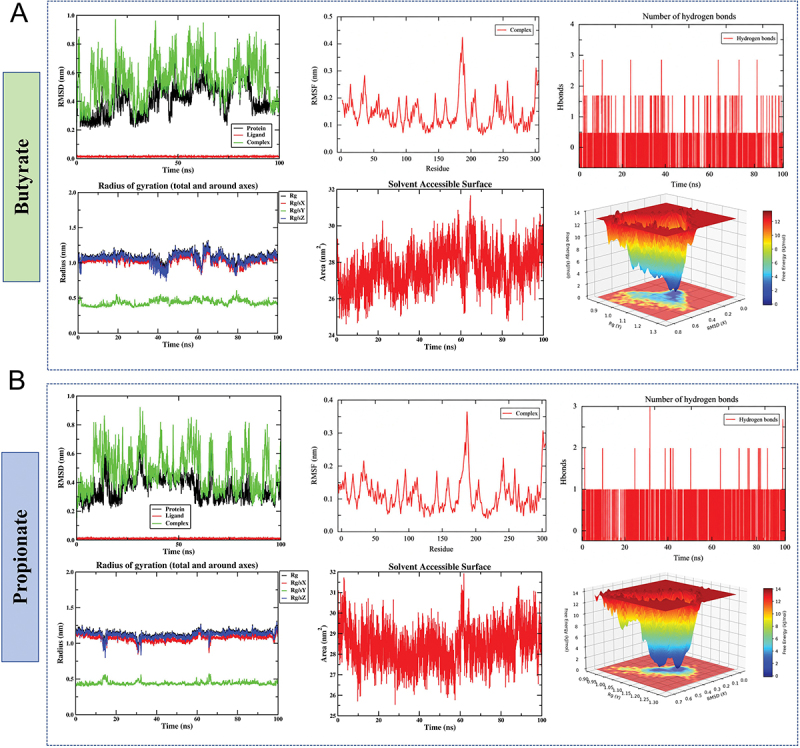
Molecular dynamics simulation results of butyrate and propionate with the JUN protein. (A) Analysis of RMSD, RMSF, hydrogen bond count, Rg, SASA, and FEL for the butyrate-JUN complex. (B) Analysis of RMSD, RMSF, hydrogen bond count, Rg, SASA, and FEL for the propionate-JUN complex. Root mean square deviation (RMSD), root mean square fluctuation (RMSF), radius of gyration (Rg), solvent-accessible surface area (SASA), free energy landscape (FEL).

The butyrate–JUN complex remained overall stable during the 100 ns molecular dynamics simulation. The backbone RMSD of JUN rapidly equilibrated and then remained within a relatively narrow range, while the complex RMSD showed moderate fluctuations without obvious destabilization, indicating overall stability with some conformational adjustment. RMSF analysis showed low flexibility for most residues, with only a few regions displaying higher fluctuations. The Rg remained stable, suggesting no major structural expansion or collapse, whereas SASA showed a slight increase, indicating mildly enhanced solvent exposure. Hydrogen bond analysis revealed that interactions between butyrate and JUN were few and transient, suggesting a weak and dynamic binding mode. The FEL showed one dominant low-energy basin together with several metastable states, indicating that the complex remained stable but retained some conformational flexibility (Figure 6(A)).

The propionate–JUN complex also showed good overall stability and appeared slightly more stable than the butyrate system. The backbone RMSD of JUN quickly reached equilibrium and remained stable, while the complex RMSD fluctuated within a relatively limited range. Most residues exhibited low RMSF values, and the Rg remained essentially constant, indicating maintained structural compactness. In contrast to the butyrate system, the SASA of the propionate complex remained more stable, suggesting less structural rearrangement. Hydrogen bonds between propionate and JUN were also limited and transient, indicating that binding was mainly maintained by weak interactions. The FEL displayed a more concentrated low-energy basin, suggesting a more confined and stable conformational state. Overall, both ligands showed weak and dynamic binding to JUN, but propionate exhibited slightly better structural stability and conformational convergence than butyrate (Figure 6(B)).

### MR analysis of gut microbiota and AKI

**Figure 7.**
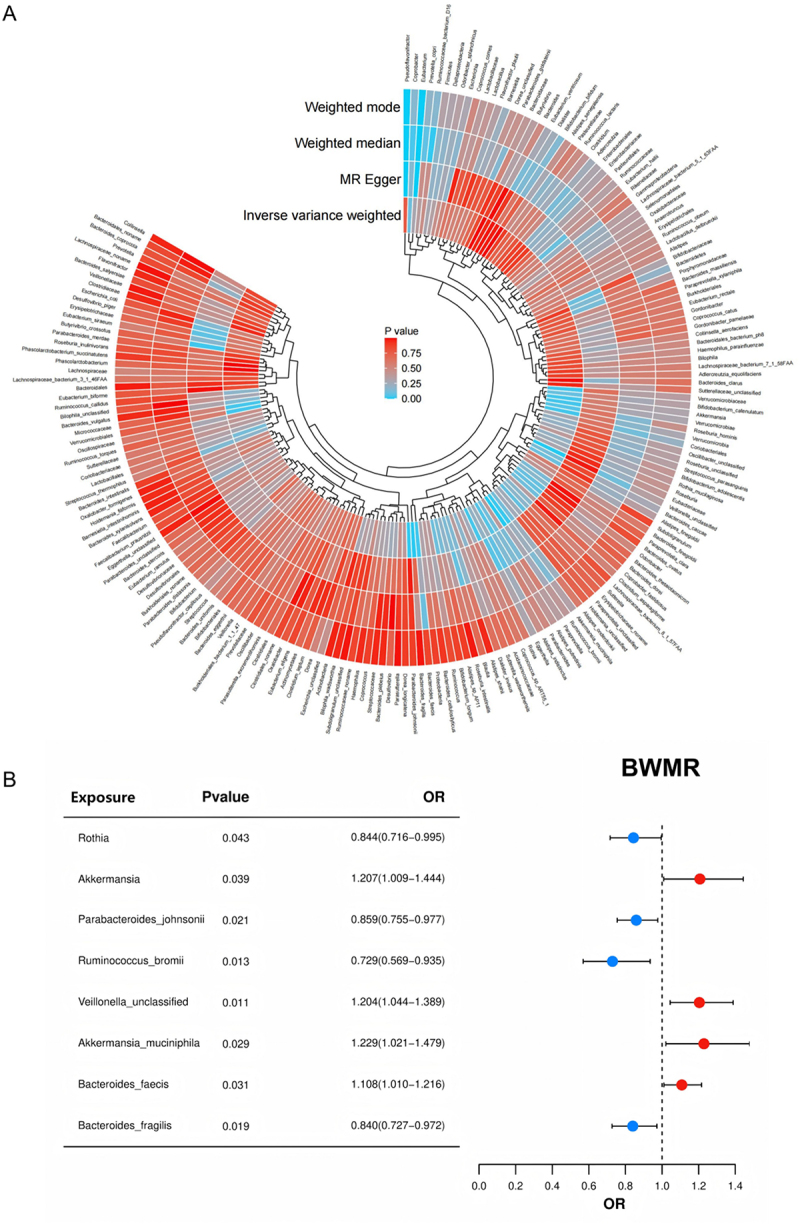
MR analysis reveals gut microbiota causally associated with renal IRI. (A) Circular heatmap demonstrating statistical significance between gut microbiota and renal IRI under four MR methods (IVW, MR-Egger, weighted median, weighted modal), colored by *p* value. (B) Forest plot of BWMR analysis identifying eight genera with significant causal association with renal IRI. Mendelian randomization, MR; Ischemia-reperfusion injury, IRI; Inverse Variance Weighted, IVW; Bayesian Weighted Mendelian Randomization, BWMR.

**Figure 8.**
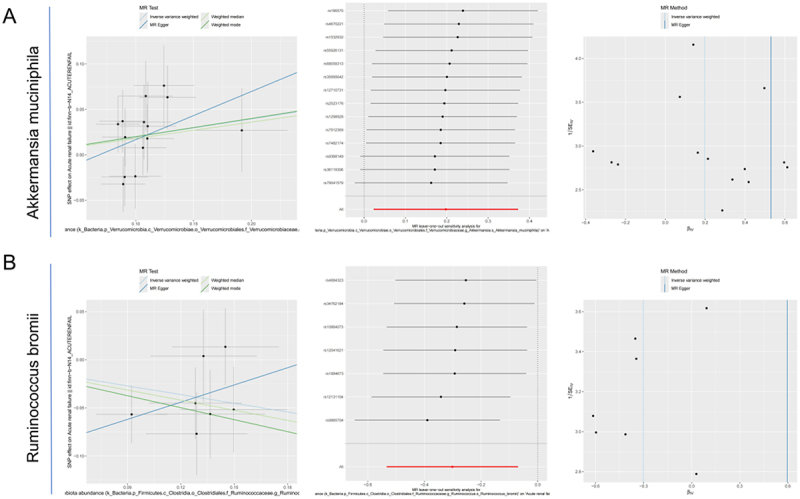
MR confirms causal associations of *Akkermansia muciniphila* and *Ruminococcus bromii* with renal IRI. (A) MR analysis for *Akkermansia muciniphila*. Left panel: scatter plot showing SNP effect estimates on exposure (x-axis) and outcome (y-axis), with regression lines from four MR methods. Middle panel: forest plot showing individual SNP causal effect estimates with confidence intervals. Right panel: funnel plot assessing potential horizontal pleiotropy. (B) MR analysis for *Ruminococcus bromii*. Mendelian randomization, MR; Ischemia-reperfusion injury, IRI; Single Nucleotide Polymorphism, SNP.

To evaluate the causal role of specific gut microbes in AKI, we conducted comprehensive MR analysis using multiple methods. A *p*-value heatmap summarizing results from IVW, MR-Egger, weighted median, and weighted mode methods is shown in Figure 7(A). Several microbial genera exhibited consistent and statistically significant associations across multiple models. Using IVW as the primary method, we identified eight genera significantly associated with kidney injury susceptibility after removing repeated genus. Positive associations were observed for *Veillonella unclassified* [*p* = 0.011, odds ratio (OR) = 1.192, 95% Confidence Interval (CI): 1.040–1.367], *A. muciniphila* (*p* = 0.026, OR = 1.219, 95% CI: 1.023–1.451), *Bacteroides faecis* (*p* = 0.031, OR = 1.101, 95% CI: 1.009–1.202), and *Akkermansia* (*p* = 0.036, OR = 1.199, 95% CI: 1.012–1.420). Conversely, *Bacteroides fragilis* (*p* = 0.011, OR = 0.844, 95% CI: 0.739–0.963), *Parabacteroides johnsonii* (*p* = 0.018, OR = 0.865, 95% CI: 0.766–0.975), *R. bromii* (*p* = 0.011, OR = 0.740, 95% CI: 0.587–0.932), and *Rothia* (*p* = 0.040, OR = 0.853, 95% CI: 0.732–0.993) were negatively associated with kidney injury susceptibility (Supplementary Table S4). BWMR validation confirmed the direction and significance of these associations (Figure 7(B)). The results of the heterogeneity test and sensitivity analysis showed that there was no heterogeneity or a horizontal polytropy in our analysis (*p* > 0.05) (Figures 8(A,B); Supplementary Table S4). Reverse MR analysis also indicated no evidence of reverse causality or horizontal pleiotropy for these taxa (Supplementary Table S5).

## Discussion

In this study, we identified a potential gut microbiota–metabolite–host regulatory axis involved in renal IRI. By integrating transcriptomic analysis, metabolite–target prediction, molecular docking, molecular dynamics simulation, binding free-energy analysis, and MR, we obtained convergent evidence supporting a pathway in which the gut microbiota-associated metabolite propionate may participate in renal inflammatory responses through JUN, with downstream involvement of the TNF and IL-17 signaling pathways. Among the microbial taxa highlighted by MR, *A. muciniphila* and *R. bromii* emerged as two key but functionally distinct commensals, both associated with propionate metabolism [24,25]. In two independent renal IRI datasets, JUN was consistently identified as an upregulated hub gene, suggesting that it may represent an important molecular interface linking microbial metabolism to host inflammatory transcriptional regulation.

A major finding of this study is the identification of JUN as the central connecting node in this integrative framework. As a core component of the AP-1 transcription factor complex, JUN regulates multiple stress-responsive programs, including apoptosis, proliferation, survival, and immune activation, in a context-dependent manner [26]. Previous studies have shown that JUN participates in both pro-inflammatory signaling and injury repair in models of ischemic cerebral infarction and myocardial injury [27,28]. In the present study, JUN was enriched in TNF and IL-17 signaling pathways, both of which are closely associated with inflammatory amplification during renal IRI. Our transcriptomic findings are also consistent with previous studies showing that ischemia–reperfusion injury is characterized by coordinated activation of inflammatory, oxidative stress, and immediate-early response programs. Movahed et al. summarized transcriptomic hallmarks of ischemia–reperfusion injury across organs and highlighted stress-response regulators and inflammatory signaling networks as central components of IRI-associated transcriptional remodeling [29]. In this context, the identification of JUN as an upregulated hub gene in our study is biologically plausible and consistent with prior evidence implicating AP-1-related signaling in ischemic injury. These findings support the view that JUN may act as a key transcriptional hub in renal inflammatory injury rather than as a simple unidirectional pro-inflammatory mediator.

Another important aspect of this study is the prioritization of propionate among candidate microbial metabolites. Although SCFAs are generally regarded as important regulators of host metabolism and immune homeostasis, their effects are highly context-dependent. At relatively low concentrations, SCFAs may promote Treg differentiation and anti-inflammatory signaling through HDAC inhibition [30–32], whereas under other conditions they may transiently activate stress-related pathways such as MAPK signaling [33,34]. In our analysis, propionate, rather than butyrate, emerged as the shared metabolite associated with the two key taxa identified by MR, namely *A. muciniphila* and *R. bromii*. On this basis, we focused subsequent analysis on the propionate–JUN relationship. Molecular docking and molecular dynamics simulation suggested favorable binding between propionate and JUN, and this interaction was further supported by molecular dynamics simulation and binding free-energy analysis, indicating that the propionate–JUN complex remained relatively stable during dynamic evaluation. Taken together, these findings strengthen the biological plausibility that propionate may participate in JUN-related inflammatory regulation during renal IRI. This interpretation is further supported by previous evidence from other ischemia–reperfusion settings. Deng et al. reported that propionate alleviated myocardial ischemia–reperfusion injury through a GPR41-dependent caveolin-1/ACE2 axis. Although derived from a different organ context, this finding supports the broader concept that microbiota-associated short-chain fatty acids may modulate ischemia-related inflammatory injury, which is consistent with the propionate-centered mechanism proposed in our study [35].

The opposite directions of association observed for *A. muciniphila* and *R. bromii* are also noteworthy. Increasing evidence suggests that gut microbial taxa should not be interpreted within a simplistic beneficial-versus-harmful framework, but rather according to their functional state and host context [36]. *A. muciniphila* has often been described as beneficial for mucosal homeostasis because it can enhance tight junction protein expression and promote mucus synthesis when the intestinal barrier is intact [37]. However, under conditions of barrier disruption or inflammatory stress, its mucus-degrading activity may expose the epithelium and facilitate TLR2/NF-κB-related inflammatory amplification [38]. In contrast, *R. bromii*, as a key resistant starch-degrading bacterium, is closely linked to SCFA-related metabolism and may contribute to immunosuppression, epithelial repair, and Treg induction through metabolites including propionate [39,40]. These observations suggest that the contribution of specific taxa to kidney injury risk depends less on static taxonomic labels than on their metabolic output, ecological context, and interactions with the host microenvironment [41–43].

From a mechanistic perspective, the propionate–JUN axis may also provide a useful framework for understanding how microbial metabolites influence transcriptional regulation. Propionate has been reported to regulate histone acetylation, including H3K9 and H3K27 modifications, through HDAC inhibition, thereby affecting chromatin accessibility and gene expression [44,45]. It is therefore possible that propionate influences JUN-related inflammatory responses not only through direct structural interaction but also through broader epigenetic regulatory mechanisms. In addition, the potential significance of this axis may extend beyond the kidney. SCFAs have been shown to attenuate MRSA-induced acute lung injury by promoting M2 macrophage polarization [46], and butyrate has been reported to improve spinal cord recovery by reducing microglial activation and neuroinflammation [47]. JUN also plays an important role in multiple forms of ischemic organ injury [48,49]. Together, these findings raise the possibility that the metabolite–transcription factor– signaling pathway framework proposed here may have broader relevance for inflammatory injury across organs. However, it should be noted that inflammatory “fluctuations” among different organs may generate asynchronous and nonlinear responses of JUN activity, and it is necessary to introduce new tools such as single-cell spatial genomics and SCFA distribution imaging to construct cross-organ signaling maps and metabolic network models, so as to advance the systematic understanding of colony-host interactions. and guide the precise application of microecological strategies in systemic inflammatory syndromes.

### Limitations and future directions

This study has several limitations. The transcriptomic analysis were based on public datasets and still require validation in additional experimental and clinical samples. In addition, because large-scale GWAS summary statistics specific to renal IRI are not yet available, the MR analysis used AKI as a proxy outcome. Although renal IRI is a major contributor to AKI and both conditions share key biological features, AKI is broader than IRI; thus, the MR findings support the relevance of the identified microbial signals to kidney injury susceptibility rather than strict specificity for renal IRI. Moreover, the metabolite–target relationships were derived from public databases and depend on current annotation coverage and prediction accuracy. Although molecular docking, molecular dynamics simulation, and binding free-energy analysis supported the plausibility and relative stability of the propionate–JUN interaction, further biological experiments are needed to determine whether this interaction acts directly on renal parenchymal cells or indirectly through systemic immune and inflammatory pathways. Despite these limitations, our study provides an integrative framework linking gut microbial signatures, microbiota-associated metabolites, host transcriptional regulation, and kidney injury susceptibility, and supports a potential gut–kidney axis involving *A. muciniphila*/*R. bromii*–propionate–JUN–TNF/IL-17 signaling in renal IRI. These findings deepen current understanding of gut microbiota-associated metabolic signals in renal inflammatory injury and highlight JUN as a potential metabolite-responsive regulatory target.

## Conclusion

In this study, we identified a novel “gut microbiota-SCFA-JUN” regulatory axis linking microbial metabolism to renal inflammatory responses. Propionate, a key metabolite, was produced by *A. muciniphila* and *R. bromii* in collaboration with their metabolisms, and targeted JUN, which realized the integration of flora signaling and host transcriptional regulation, and provided a new theoretical basis and targeting direction for microcosmic interventions in renal IRI, and JUN could become a metabolism-sensitive precision therapeutic target. metabolism-sensitive precise therapeutic target.

## Supplementary Material

supplementary material.xlsx

## Data Availability

The dataset supporting this study is openly available in FigShare at https://doi.org/10.6084/m9.figshare.29360795 [50].
